# Prevalence of Overweight and Obesity and Their Impact on Lung Function in Healthy Bulgarian Children and Adolescents: A Cross-Sectional Study

**DOI:** 10.3390/pathophysiology33030050

**Published:** 2026-07-14

**Authors:** Meri Hristamyan, Stoilka Mandadzhieva, Plamena Stoimenova, Nikolay Mandadzhiev, Blagoi Marinov

**Affiliations:** 1 Department of Epidemiology and Disaster Medicine, Faculty of Public Health, Medical University of Plovdiv, 4002 Plovdiv, Bulgaria; 2 Department of Pathophysiology, Faculty of Medicine, Medical University of Plovdiv, 4002 Plovdiv, Bulgaria; 3 Department of Physiology, Faculty of Medicine, Medical University of Plovdiv, 4002 Plovdiv, Bulgaria

**Keywords:** children, pulmonary function testing, overweight, obese, prevalence

## Abstract

**Background/Objectives**: There is increasing evidence on the effect of obesity on lung function in children with and without respiratory diseases. The aim of was to evaluate the prevalence of overweight and obesity and their impact on lung function parameters among healthy children. **Methods**: Six hundred and seventy-one healthy Bulgarian school children (339 males) aged 7–18 years were included in the study. All participants completed anthropometric measurements, including standing height, weight, and BMI. The studied group underwent comprehensive pulmonary function assessment. **Results**: The overweight and obese children group consisted of 131—overweight, 97, and obese, 34, children, accounting for 19.5% or every fifth child. The highest prevalence of overweight was at the ages of 10, 11, 12 and 17 years, and that of obesity was 7, 10 and 14 years, respectively. We compared normal weight, overweight and obese children in every age group and found that an increase in weight was associated with increased height and increased lung function parameters, such as FVC, FEV_1_, PEF, and FEF_50_. To overcome the effect of height, we compared normal weight and overweight obese children in height groups and found nonsignificant differences in the spirometric indices. Only in the 130–139 cm group the obese children had lower values of FEV_1_(L): 1.78 ± 0.13 vs. 1.91 ± 0.19 (NS) in children with normal weight. **Conclusions**: The increase in weight is associated with height growth, and the same pattern is observed for the mean spirometry indices, with lowest values found only in obese 7-year-old children.

## 1. Introduction

The complex assessment of respiratory functions among healthy children includes comprehensive measurements of anthropometric parameters. The main determinants of lung function are gender, age, height, and weight, and they contribute to the calculation of the predicted normal values. There is increasing evidence on the effect of obesity on lung function in children with and without respiratory diseases [1,2,3]. Obesity is a rapidly worsening global health crisis, but its detrimental impact on the respiratory system remains frequently overlooked [4].

The increasing prevalence of overweight and obesity among children has emerged as a critical public health challenge globally, with significant implications for respiratory health and increased risk for pulmonary issues [5]. Obesity-related inflammation and respiratory problems can also lead to further cardiometabolic risks [5,6]. In the WHO European Region, nearly one in three children aged 7–9 years are affected by excess weight [7], whereas global estimates indicate that obesity rates tripled in children and adolescents between 1990 and 2021 [8]. Bulgaria reflects this trend, with studies demonstrating a concerning increase from 28.2% overweight/obesity prevalence in 2008 to 32% in recent years among pediatric populations [9]. This increase has been attributed to nutritional transitions, reduced physical activity, and socioeconomic factors [10].

Height, weight, and body mass index (BMI) remain the most commonly used anthropometric variables in clinical and research settings to evaluate growth and lung function in pediatric populations [11]. In recent years, there has been increasing evidence on the effects of overweight and obesity on lung function in children, including those without respiratory diseases [12,13,14].

Although obesity is not part of most predictive models, it can still influence measured lung function through mechanical constraints (chest wall loading, reduced functional residual capacity), systemic inflammation, and altered chest wall mechanics, which are not captured by standard prediction equations [15].

These parameters are routinely incorporated into spirometric reference equations to establish predicted values for lung volumes and flows [16]. They include SVC, FVC, FEV_1_, PEF, and FEF_50_, which are the principal spirometric outcomes considered in this study [17,18,19,20,21].

Scientific studies have shown that FEF_50_ and FEF_25–75%_ are highly correlated with a stable ratio and generally provide similar information about small airway function [22]. The correlation coefficient between them is very high (r^2^ ≈ 0.96) [23], and because of this, some experts suggest that reporting both is redundant and that FEF_50_ alone is sufficient. (21) In children, especially those who are overweight or obese, these lung function parameters are valuable for detecting early changes in airway function [24,25]. One study revealed that overweight and obesity at approximately 7 years of age were associated with a decreased FEV_1_/FVC ratio and decreased FEF_50_ (maximal expiratory flow at 50% of FVC, equivalent to FEF_50_) at 12 years of age [26]. Other researchers state that reductions in FEV_1_/FVC ratio and mid-expiratory flows, such as FEF_50_ or FEF_25–75%_, may indicate early small airway obstruction or dysanapsis, even when FEV_1_ and FVC are normal [27]. A systematic review summarizes multiple studies showing that obesity in children is associated with decreased values of FEF_50_ (forced expiratory flow at 50% of FVC), along with other spirometric parameters such as FEV_1_, FVC, and FEV_1_/FVC, and states that the decrease in FEF_50_ supports the presence of small-to-medium-airway impairment related to obesity [28]. Emerging research from the BAMSE cohort (n = 3200) revealed that persistent childhood obesity leads to adult lung function impairment through multiple pathways: mechanical restriction from visceral fat, chronic low-grade inflammation, and altered chest wall mechanics [29]. On the other hand, transient childhood obesity with subsequent weight normalization has no residual pulmonary effects [29]. These findings highlight the importance of early lifestyle interventions aimed at mitigating long-term respiratory consequences, since normalizing BMI before puberty can reverse obesity-associated lung function deficits [10,30].

In Bulgaria, the prevalence of overweight and obesity among children has been steadily increasing [9]. Despite this, there are limited data specifically exploring how elevated BMI influences lung function parameters in healthy Bulgarian children. Given that height, weight, and BMI are key determinants of lung function prediction equations, it is crucial to understand their relationship with pulmonary outcomes in this population to improve clinical assessment and public health strategies.

The aim of the present study was to evaluate the prevalence of overweight and obesity and their impact on lung function parameters among healthy Bulgarian children aged 7–18 years.

## 2. Materials and Methods

### 2.1. The Study Design and Characteristics of the Participants

This retrospective cross-sectional analysis included six hundred and seventy-one healthy Bulgarian school children (339 males and 332 females) aged 7–18 years. All tested subjects were residents of Plovdiv, a city in southern Bulgaria, with an average altitude of 150 m.

Following initial ethical approval, this study evaluated data collected over the period of 2013–2023, excluding the period of 2020–2022 due to the COVID-19 pandemic. While standard pediatric reference models (such as the Global Lung Function Initiative [GLI]) establish predicted values based on age, sex, height, and ethnicity without utilizing weight, body weight was recorded and entered as it was a mandatory field required for patient profile initialization by our laboratory spirometric software (Master Screen Diffusion, JAEGER/CareFusion, Germany with SentrySuite software (version 2.7).

The study participants were healthy Bulgarian school children from two schools in Plovdiv, South Bulgaria, and all tests were performed in the certified laboratory at Medical University of Plovdiv, a single research center. The PFT laboratory is equipped with a complex spirometric system, applying ERS/ATS standards, and the measurements were done by a certified technician.

Written informed consent was obtained from the parents or guardians of all participants before the start of the test procedures. All methods were performed in accordance with the ethical standards of the Declaration of Helsinki.

**Figure 1:**
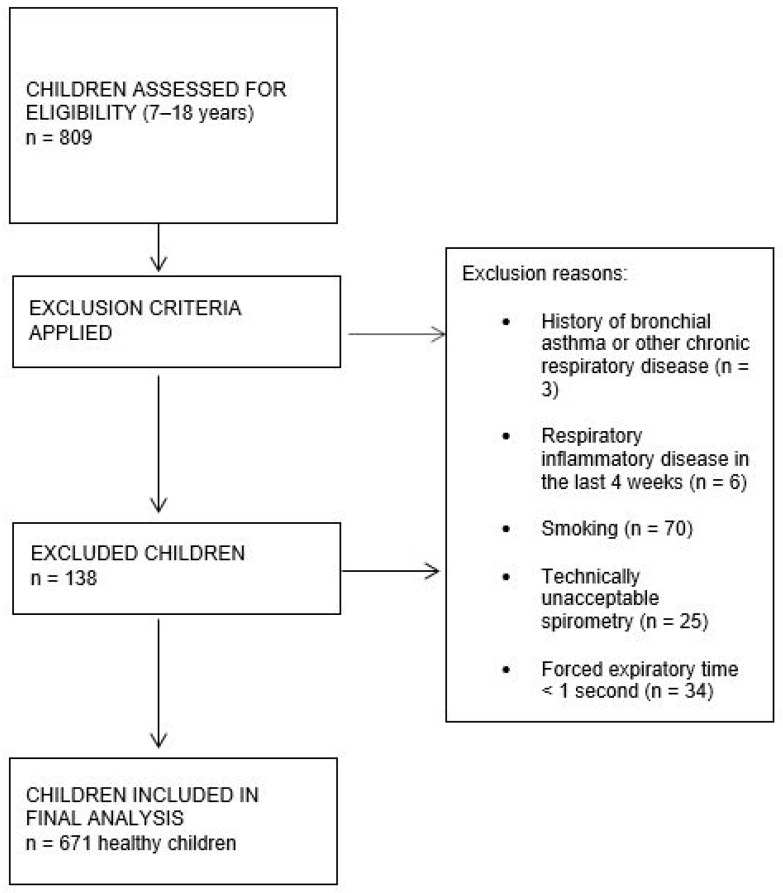
Study flowchart of participant recruitment and selection.

The initial study included 809 children (389 males and 420 females), and after spirometry tests, all participants who met the following exclusion criteria were excluded from the analysis: current or past medical history of asthma or other chronic respiratory conditions; respiratory infection in the last four weeks; unacceptable spirometry testing; and forced expiratory time under 1 s (Figure 1).

The final dataset included 671 healthy Bulgarian school children (339 males and 332 females) aged 7–18 years.

### 2.2. Anthropometric Measurements

All participants completed comprehensive anthropometric measurements. Standing height and body weight were measured in all children without wearing shoes. Standing height was measured with a combined stadiometer Seca (Seca GMBH & Co., Hamburg, Germany) via a standardized technique to the nearest cm. The same device was used to measure body weight to the nearest kg. For all studied subjects, body mass index (BMI) was calculated.

### 2.3. Defining Overweight and Obesity

For each child, BMI (kg/m^2^) was calculated and classified according to the cutoff points published by Cole, 2000, for each gender and age between 7 and 18 years [31].

We performed also measurements of skinfold thickness over two parts of the right side of the body—over the triceps and subscapular regions—using a Harpenden caliper (Harpenden, British indicators, Burgess Hill, West Sussex, UK) to calculate the percent body fat. Every measurement was repeated three times, and the average value was used. All average values of the skinfolds were added together, and the sum was used in the Slaughter equations to calculate the fat-free mass and, respectively, the percent body fat [32].

### 2.4. Spirometric Measurements

The studied group underwent pulmonary function assessment (Master Screen Diffusion, Jaeger, Wuerzburg, Germany) in a certified laboratory applying the ERS/ATS guidelines to ensure quality [33].

All spirometry testing was performed in the morning and before lunch time. All children were asked to avoid drinking beverages, having a heavy meal before the examination and all children were resting for at least 15 min before the PFT measurements. All children in this dataset were nonsmokers. The respiratory parameters that were measured were SVC, FVC, FEV_1_, FEV_1_/FVC, PEF, FEF_50_ and the respective % predicted and there were included in the analysis.

Regular calibration was performed with a 3 L calibration syringe twice daily. The equipment was suitable for pediatric assessment, and all technicians were well trained to collaborate with children. Pulmonary function testing was performed in the sitting erect body position wearing a nose clip in the following steps:

- Slow vital capacity;
- Forced spirometry.

All spirometry maneuvers were explained in small groups, and every child was also instructed individually prior to each test. To obtain forced spirometry parameters, every child was asked to exhale as deeply as possible and then breathe in to the total lung capacity and immediately start rapid and forceful exhalation to the residual volume for at least 6 s.

Forced spirometry measurements met the general acceptability criteria for the start of the test during, and the end of the test. Every child was asked to repeat the measurements until three technically acceptable flow–volume curves were recorded. The largest FVC and FEV_1_ were selected from these technically acceptable and repeatable attempts.

Some young children aged less than 8 years were unable to produce forced expiratory time longer than 1 s, making it impossible to record FEV_1_ values. All these participants were excluded from the final dataset.

Spirometry quality assurance included examination of test values and evaluation of both the volume–time and flow–volume curves for evidence of technical errors. During testing, technicians recorded only valid tests, composed of at least 3 acceptable maneuvers with repeatable values for both FVC and FEV_1_.

Repeatability during testing was ensured by difference between the largest and second largest values for both FVC and for FEV1 within 0.15 L (150 mL) and in children under 10 years 0.10 L (100 mL) [33]. Maneuvers were attempted, up to a maximum of 8, to meet these criteria for a valid test. Repeatability was ensured by including 3 maximal effort curves of the same size and shape.

### 2.5. Statistical Methods

The results were statistically analyzed using SPSS software, version 20.0. Descriptive statistics were used in the analysis, including methods of frequency, variation, correlation, nonparametric tests (in cases of uneven data distribution), and graphical analyses to explore the relationships between spirometry and anthropometric variables. The results are expressed as means ± standard deviations (means ± SDs). One-way analysis of covariance (ANCOVA) was performed to investigate the effects of overweight/obesity on respiratory indices, adjusting for gender, age, and height; post hoc analysis was conducted using Bonferroni correction. Stepwise regression analysis was used to determine most important predictors for FVC and FEV_1_. The variables entered into the models included age, sex, height, BMI category, and percent body fat derived from skinfold measurements. Differences were considered statistically significant if the *p* value was less than 0.05.

### 2.6. Ethics

All participants above the age of 16, their parents or legal guardians in the case of children under 16, fulfilled informed consent prior to participating in the study. The study protocol was reviewed and approved by the Ethics Committee at the Medical University of Plovdiv, Bulgaria (Protocol №1/15. 03. 2012). The study was conducted in accordance with the principles outlined in the Declaration of Helsinki and all applicable national regulations concerning research involving human subjects.

## 3. Results

**Figure 2:**
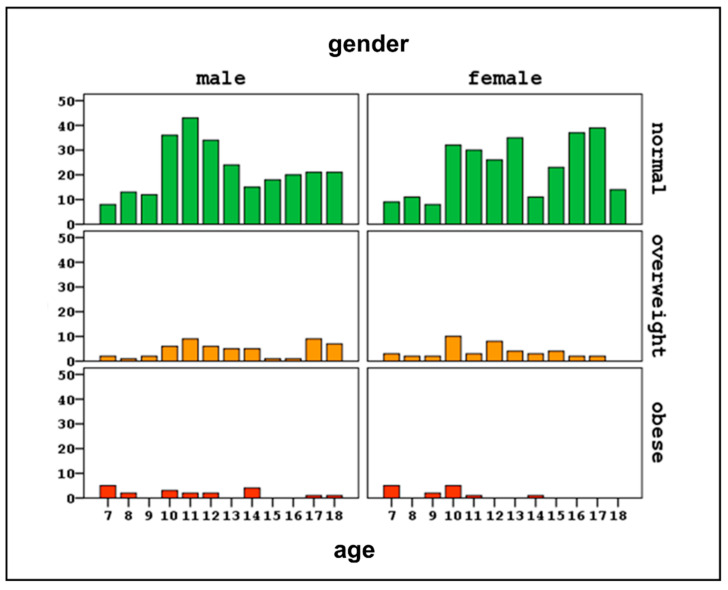
Prevalence of overweight and obesity in the studied group.

We used Cole (2000) [31] reference values to evaluate the prevalence of overweight and obesity in the studied population for every gender and age group. Among all 671 children aged 7–18 years included in the study, the overweight and obese group consisted of 131 children, overweight—97 and obese—34, accounting for 19.5% or every fifth child. The highest prevalence of overweight was at the ages of 10, 11, 12, and 17 years, and obesity—respectively—at 7, 10, and 14 years, as presented in Figure 2.

The spirometric measurements of the three BMI-related groups of children are shown in Table 1. One-way analysis of variance revealed that the effect of BMI on the measured absolute values of FVC and VC was statistically significant, and % predicted VC. Post hoc analysis indicated that all the above parameters were reduced in overweight or obese children compared to children with normal weight, but not to a statistically significant degree. As expected, similar results were obtained in the studied pediatric population when we analyzed the values of the spirometric variables adjusted for gender, age, and height by one-way analysis of covariance, the results of which are presented in Table 1.

The number of obese children is relatively small, so we performed subgroup analyses by sex to examine effects on respiratory parameters. Obese children were more common in the younger age range (up to approximately 10–11 years), with no significant sex difference. Notably, both weight and height were higher in these children. Since height is the main determinant of lung function, the spirometric values in these healthy children remained within normal reference ranges.

In girls, BMI category was significantly associated with FVC (F(2,327) = 6.307, *p* = 0.002), FEV_1_ (F(2,327) = 3.157, *p* = 0.044), and FEF_50_ (F(2,327) = 3.092, *p* = 0.047) after adjustment for age and height. No significant association was observed between BMI category and the FEV_1_/FVC ratio (F(2,327) = 0.462, *p* = 0.631). Height was the strongest independent predictor of pulmonary function across all models.

In contrast to boys, among whom BMI category was not significantly associated with any spirometric parameter, girls demonstrated significant associations between BMI category and FVC, FEV_1_, and FEF_50_ after adjustment for age and height. However, BMI was not associated with the FEV_1_/FVC ratio in either sex.

We compared normal weight, children with overweight and obese children in every age group and found that the increase in weight was associated with height growth and the same pattern of increase in the following lung function parameters—VC, FVC, FEV_1_, PEF, and FEF_50_. For example, at the age of 10, children with normal weight had mean height of 142.1 ± 6.6, vs. 147.8 ± 6.1 (*p* < 0.05) in overweight and 151.6 ± 11.1 (*p* < 0.05) in obese children. The same model was found in this age group also in FEV_1_(L): children with normal weight had mean value of 2.16 ± 0.31, vs. 2.37 ± 0.28 (*p* < 0.05) in overweight and 2.71 ± 0.54 (*p* < 0.05) in obese children, respectively.

**Figure 3:**
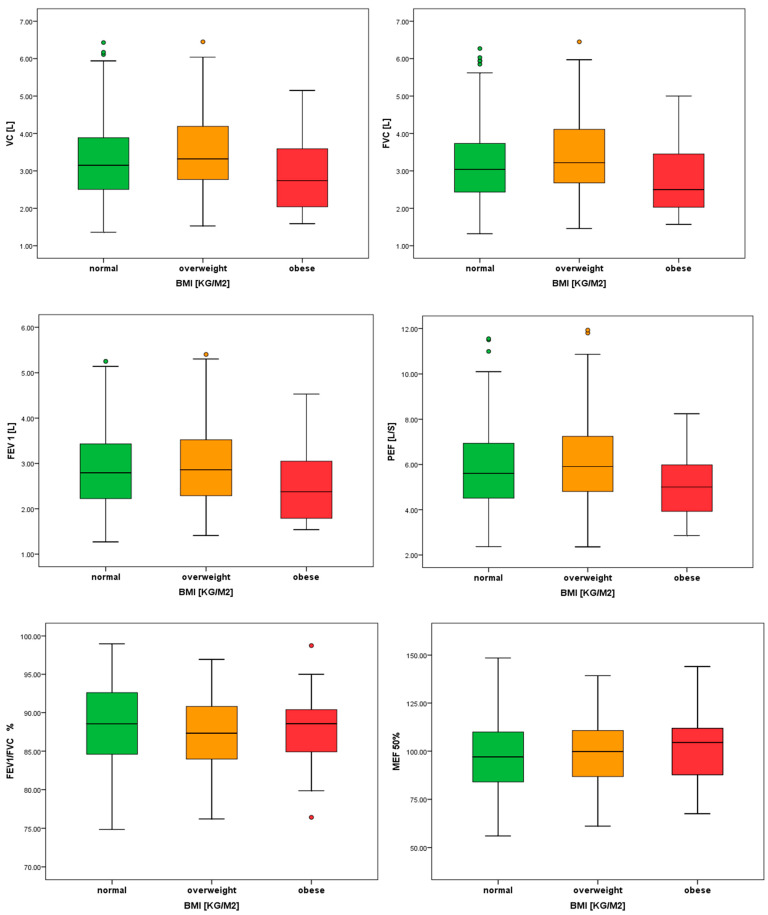
Effects of BMI on the absolute values of VC, FVC, FEV_1_, and PEF and the % predicted of FEV_1_/FVC ratio and % predicted of FEF_50_.

Figure 3 shows the effects of BMI on the absolute values of VC, FVC, FEV_1_, and PEF and the % predicted of FEV_1_/FVC ratio and the % predicted of FEF_50_ in all studied children.

Individuals with obesity had the lowest values for VC, FVC, FEV_1_ and PEF, but a significant difference was found only between the overweight and obesity groups *p* = 0.005 for VC, and *p* = 0.008 for FEV_1_. The obese participants had similar FEV_1_/FVC values, and there were no group differences in the percentage predicted FEV_1_, PEF or FEF_50_.

**Figure 4:**
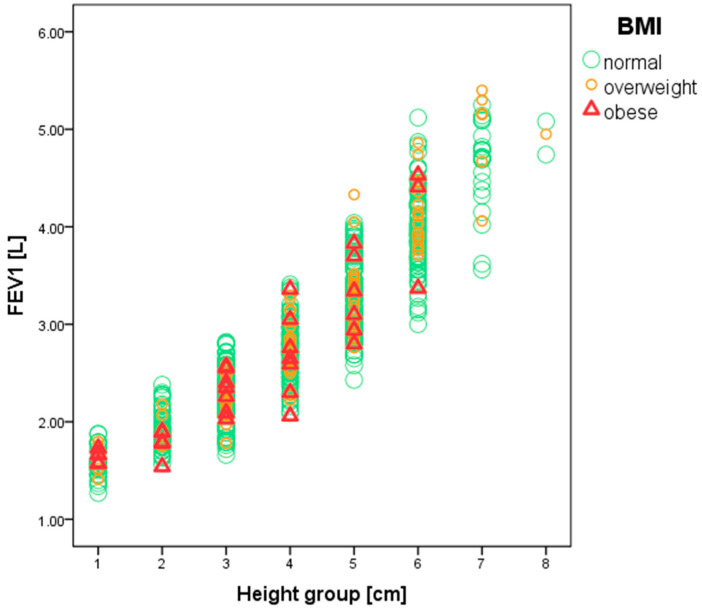
Scatter plot of FEV_1_ vs. height groups in normal weight, overweight and obese children. Legend: 1 = 120–129 cm; 2 = 130–139 cm; 3 = 140–149 cm; 4 = 150–159 cm; 5 = 160–169 cm; 6 = 170–179 cm; 7 = 180–189 cm; 8 = over 190 cm.

The most important predictor of respiratory parameters was height, and to overcome the effect of height we divided the studied population into 10 cm height groups. Among these height groups, we compared normal weight, overweight and obese children and found nonsignificant differences in the main spirometric indices. Compared with normal weight children, obese children in the 130–139 cm group presented lower values of FEV_1_(L): 1.78 ± 0.13 vs. 1.91 ± 0.19 (NS) and similar pattern was found in the 150–159 cm group (Figure 4).

Pulmonary function parameters correlated positively and to varying degrees with age and anthropometric indicators.

A multiple linear regression analysis was performed in 671 children (ages 7–18) examined how overweight and obesity relate to lung function (FVC and FEV_1_), while adjusting for age, sex, and height.

For FVC, all variables were significant predictors, with height having the strongest effect, followed by age and sex. Height demonstrated the strongest relative impact on FVC variation (beta = 0.755, *p* < 0.001), followed by age (beta = 0.186, *p* < 0.001) and sex (beta = −0.147, *p* < 0.001). After adjustment, overweight and obesity still showed a small but statistically significant positive association with FVC: (B = 0.085, beta = 0.045, *p* = 0.001). Specifically, being overweight was associated with an average increase in FVC of 0.085 L (85 mL)—a finding that is frequently attributed to accelerated somatic growth and larger thoracic size relative to their normal-weight peers.

For FEV_1_, the same pattern of covariates was observed, with height again the dominant predictor of FEV_1_ (beta = 0.785, *p* < 0.001), followed by age (beta = 0.167, *p* < 0.001) and sex (beta = −0.101, *p* < 0.001). Overweight and obesity also had a smaller positive effect on FEV_1_: (B = 0.048, beta = 0.029, *p* = 0.029). Being overweight was associated with a modest increase in FEV_1_ of 0.048 L (48 mL).

Overall, both lung function measures increased slightly in overweight and obese children even after adjustments, with the increase being larger for FVC than FEV_1_. This slightly greater increase in FVC compared with FEV_1_ may reflect differential airway-parenchymal growth. However, the present study was not designed to assess dysanapsis directly, as no airway size indices were measured and no significant differences in FEV_1_/FVC were observed.

## 4. Discussion

According to the WHO, as of 2022, over 2.5 billion adults, over 390 million children and adolescents aged 5–19 years and 37 million children under the age of 5 are overweight [34]. Additionally, more than one billion people worldwide are living with obesity, including 159 million children and adolescents aged 5–19 years [34]. Globally, childhood overweight and obesity rates are projected to reach 30.0% by 2030 (34.2% for boys, 27.4% for girls) [35]. A systematic review and meta-analysis of 2033 studies from 154 countries revealed that the overall prevalence of obesity in children and adolescents was 14.8% and 22.2%, respectively [36]. In many European countries, one in three school-aged children (approximately 33%) are living with overweight or obesity [36,37]. Eastern and northern Europe are among the subregions with the highest prevalence [38]. In the U.S., as of 2017–2020, approximately 19.7% of children and adolescents aged 2–19 years were obese, and 16.1% were overweight, totaling around 36% with overweight or obesity combined [39]. These weight issues are most prevalent among adolescents, followed by school-age children, and are least prevalent among preschoolers.

A 2022 cross-sectional study of 1034 children (aged 6–13) in North Macedonia [40] revealed that 33% were overweight (19.5%) or obese (13.5%). Boys had a higher prevalence (37.1%) than girls did (29.1%). The overweight prevalence increased with age, from 5.8% at 6–7 years of age to 19.5% at 12–13 years, with a marked increase in overweight and obesity between the ages of 6–7 and 8–9 years, possibly linked to the onset of puberty. Our overall prevalence of overweight and obesity is lower than that in recent North Macedonian and US reports, but the age-related trends are consistent. With respect to lung function parameters, several recent studies have confirmed that overweight and obese children tend to have higher absolute lung function values (FVC, FEV_1_, PEF) compared to normal-weight children, most likely due to the fact that they are also taller and larger in body size [41,42,43]. For example, the EXAMIN YOUTH study revealed that overweight and obese children have significantly higher FEV_1_, FVC, and PEF than their normal-weight peers, but this difference is explained by their greater height and advanced growth [42]. A study on obese Thai children and adolescents reported that among the 45 obese children/adolescents (mean age 11.9 years) included in their study, 73.2% had abnormal lung function; the most common abnormality was decreased Functional Residual Capacity (FRC) (64.4%) [44]. In the present study, the FEV_1_/FVC ratio was found to be lower in obese children and adolescents, specifically in some height groups, which is consistent with literature findings of lower ratios in obese children, indicating airflow limitation [1,2]. After matching for height, differences in spirometric indices between normal-weight and overweight/obese children in the present study became nonsignificant, except for a nonsignificant trend toward lower FEV_1_ in obese children in the 130–139 cm group. Other authors reported that after adjusting to height, most differences in FVC and FEV_1_ between weight groups disappeared. The PIAMA cohort and other European studies reported higher FVC and FEV_1_ in children with high BMI, but highlighted that the apparent advantage in lung function among overweight/obese children is due to height; after adjustment, lung function is similar or may even be lower in obese children, particularly for FEV_1_/FVC ratio [45,46,47].

One possible reason for the higher prevalence of overweight and obesity among boys, particularly after 14 years of age, is the sex-specific pattern of pubertal growth, and since during puberty boys typically gain more fat-free mass and muscle mass than girls, the increased BMI may reflect not only adiposity but also a larger lean body compartment, which is positively related to lung volumes and forced expiratory indices [48,49,50]. This may also explain why some spirometric parameters differed significantly only between the overweight and obese groups, but not between obese and normal-weight children, since BMI does not differentiate fat from muscle, and the respiratory impact of excess body weight may become more apparent only when adiposity is substantial or when the balance shifted [51].

Our findings suggest a sex-specific effect of overweight and obesity on pulmonary function. While no independent association was observed in boys, BMI category was associated with lung volumes (FVC and FEV_1_) and expiratory flow at 50% of forced vital capacity (FEF_50_) in girls. The absence of an association with the FEV_1_/FVC ratio indicates that obesity-related changes are more likely related to lung volumes and airway mechanics rather than airflow obstruction.

Overweight children in our study were also taller, and height is one of the strongest determinants of lung function [52,53]. Therefore, the higher values of some spirometric parameters in overweight children probably reflect greater body size and earlier pubertal growth rather than a true functional advantage of excess weight [54]. This pattern may be particularly evident in children who are entering the pubertal growth spurt, when height and lung volumes increase in parallel [55].

Our findings of increased FEF_50_ before height adjustment align with increased FVC and FEV_1_ in a Brazilian report [56], but the literature suggests that small airway flows like FEF_25–75%_ and FEF_50_ tend to be reduced or impaired in obese individuals, especially when fat mass is considered [1,2,57]. Changes in waist circumference inversely affect FEF_25–75%_, indicating worsening small airway function with increased abdominal adiposity [30]. Our study did not analyze waist circumference or fat distribution, which may explain why no significant impairment in FEF_50_ after height adjustment was found. Gonzalez-Barcala et al. [58] included in their study 2408 healthy children and adolescents aged 6–18 years and showed that the increase in fat mass (FM) had a negative effect on respiratory functions in both sexes, stronger in boys.

### Study Limitations

This study has several limitations. First, the retrospective cross-sectional design precludes causal inference and does not allow assessment of longitudinal changes in growth or lung function. Second, the sample was drawn from healthy schoolchildren in a single city in Bulgaria, which may limit generalizability to other pediatric populations. Third, the absence of direct measures of central adiposity, such as waist circumference, and the lack of more detailed body composition data limit our ability to distinguish the independent contributions of fat mass and fat-free mass to pulmonary function. In addition, no direct indices of airway size or dysanapsis were available, which restricts mechanistic interpretation of the spirometric findings. Finally, the obese group was relatively small compared with the normal-weight and overweight groups, which may have reduced statistical power for some comparisons. Future studies should use prospective designs, include larger and more balanced samples, and incorporate more comprehensive measures of adiposity and airway structure.

## 5. Conclusions

The prevalence of overweight and obesity in healthy Bulgarian children was 19.5%. The increase in weight was accompanied by greater height and similar increases in mean spirometry indices, indicating that higher lung function values in overweight and obese children are largely explained by their larger body size. After adjusting for height, differences in lung function between weight groups were minimal, suggesting that excess weight alone does not significantly impact spirometric parameters. Notably, lower FEV_1_ values were observed only in obese 7-year-old children, highlighting the importance of early monitoring and intervention in this age group.

## Figures and Tables

**Table 1:** Main spirometric parameters in the studied group (n = 671).

Parameter	Normal n = 540	Overweight n = 97	Obese n = 34	*p* Value
VC (L) *	3.20 ± 0.94	3.44 ± 1.05	2.83 ± 0.90	0.007
VC % pred.	101.06 ± 9.52	103.2 ± 11.01	101.00 ± 9.35	0.008
FVC (L) *	3.10 ± 0.93	3.35 ± 1.05	2.71 ± 0.86	0.002
FVC % pred.	101.52 ± 15.95	101.87 ± 11.19	99.26 ± 9.89	NS
FEV_1_(L) *	2.82 ± 0.82	2.96 ± 0.85	2.46 ± 0.74	NS
FEV_1_% pred.	107.77 ± 10.16	108.26 ± 10.64	107.39 ± 9.84	NS
FEV_1_/FVC %	88.49 ± 5.34	86.83 ± 5.18	87.70 ± 4.67	NS
PEF (L/s) *	5.69 ± 1.65	6.03 ± 1.83	4.98 ± 1.37	NS
PEF% pred.	98.57 ± 15.30	101.03 ± 16.93	96.94 ± 12.82	NS
FEF_50_ (L/s) *	3.52 ± 1.06	3.67 ± 0.99	3.28 ± 0.96	NS
FEF _50_% pred.	97.94 ± 18.28	99.22 ± 17.69	101.82 ± 17.98	NS

Legend: VC, vital capacity; VC % pred., vital capacity as percentage of predicted value; FVC, forced vital capacity; FVC % pred., forced vital capacity as percentage of predicted value; FEV1, forced expiratory volume in one second; FEV1% pred., forced expiratory volume in one second as percentage of predicted value; FEV_1_/FVC %, ratio of FEV_1_ to FVC expressed as percentage; PEF, peak expiratory flow; PEF % pred., peak expiratory flow as percentage of predicted value; FEF_50_, forced expiratory flow at 50% of vital capacity; FEF_50_% pred., forced expiratory flow at 50% of vital capacity as percentage of predicted value. All data are presented as mean ± SD. * Parameters marked with an asterisk were adjusted for gender, age, and height using ANCOVA. NS = not significant.

## Data Availability

The data presented in this study are available on request from the corresponding author.

## Abbreviations

The following abbreviations are used in this manuscript:

BMI	Body Mass Index
VC	Vital Capacity
FEV_1_	Forced Expiratory Volume in 1 s
FVC	Forced Vital Capacity
PEF	Peak Expiratory Flow
FEF_50_	Forced Expiratory Flow at 50% of FVC
FEF_25–75%_	Forced Expiratory Flow between 25% and 75% of FVC
MEF50	Maximal Expiratory Flow at 50% of FVC (equivalent to FEF_50_)
ERS	European Respiratory Society
ATS	American Thoracic Society
Tlco	Diffusion Capacity for Carbon Monoxide (transfer factor)
SPSS	Statistical Package for Social Sciences
WHO	World Health Organization
IOTF	International Obesity Task Force
FRC	Functional Residual Capacity
